# Optimizing the procedure of grain nutrient predictions in barley via hyperspectral imaging

**DOI:** 10.1371/journal.pone.0224491

**Published:** 2019-11-07

**Authors:** Mathias Wiegmann, Andreas Backhaus, Udo Seiffert, William T. B. Thomas, Andrew J. Flavell, Klaus Pillen, Andreas Maurer

**Affiliations:** 1 Martin Luther University Halle-Wittenberg (MLU), Institute of Agricultural and Nutritional Sciences, Chair of Plant Breeding, Halle, Germany; 2 Fraunhofer Institute for Factory Operation and Automation (IFF), Magdeburg, Germany; 3 The James Hutton Institute (JHI), Invergowrie, Dundee, Scotland, United Kingdom; 4 University of Dundee at JHI, School of Life Sciences, Invergowrie, Dundee, Scotland, United Kingdom; University of Sao Paulo/Luiz de Queiroz Agriculture College, BRAZIL

## Abstract

Hyperspectral imaging enables researchers and plant breeders to analyze various traits of interest like nutritional value in high throughput. In order to achieve this, the optimal design of a reliable calibration model, linking the measured spectra with the investigated traits, is necessary. In the present study we investigated the impact of different regression models, calibration set sizes and calibration set compositions on prediction performance. For this purpose, we analyzed concentrations of six globally relevant grain nutrients of the wild barley population HEB-YIELD as case study. The data comprised 1,593 plots, grown in 2015 and 2016 at the locations Dundee and Halle, which have been entirely analyzed through traditional laboratory methods and hyperspectral imaging. The results indicated that a linear regression model based on partial least squares outperformed neural networks in this particular data modelling task. There existed a positive relationship between the number of samples in a calibration model and prediction performance, with a local optimum at a calibration set size of ~40% of the total data. The inclusion of samples from several years and locations could clearly improve the predictions of the investigated nutrient traits at small calibration set sizes. It should be stated that the expansion of calibration models with additional samples is only useful as long as they are able to increase trait variability. Models obtained in a certain environment were only to a limited extent transferable to other environments. They should therefore be successively upgraded with new calibration data to enable a reliable prediction of the desired traits. The presented results will assist the design and conceptualization of future hyperspectral imaging projects in order to achieve reliable predictions. It will in general help to establish practical applications of hyperspectral imaging systems, for instance in plant breeding concepts.

## Introduction

Cereals form the basis of human nutrition all over the world, since they provide us with our daily food [1,2]. Their grains do not only contain energy in form of carbohydrates, but also proteins, fiber and nutrients [3–6]. They represent a source for processed food products like wheat flour for baking [7] and barley malt used in the beverage industry [6,8]. Moreover, cereals supply livestock breeding with fodder, which has specific quality requirements for animal nutrition [9,10].

Barley (*Hordeum vulgare* ssp. *vulgare*) is one of these cereals and the world’s fourth most important cereal crop regarding production [8,11]. It serves mainly as source for fodder, malt and food [6,8]. In each of these uses, barley and processed barley products need to meet prescribed quality requirements [12–14]. In this regard the protein concentration of mature grains defines if barley can be used for malt (10–12% grain raw protein concentration) or fodder (no restrictions) production [12,15]. Another example would be the mineral content or rather nutritional value of barley grains, which is important if humans or animals consume barley. For example, about one billion people suffer from low intakes of proteins and nutrients, especially iron, zinc and calcium [16–18].

The majority of grain quality measurements is based on wet chemistry analysis, like the determination of the nutritional value of seeds or the digestibility of animal fodder. The results obtained from these techniques are precise and trustworthy, however the methods themselves are time-consuming, labor-intensive and expensive [19–21]. In addition, in most cases they are destructive, i.e. the plant material (e.g. seeds) is destroyed during the analysis. These drawbacks prevent the standardized application of quality analysis of high numbers of genotypes in breeding programs, especially in early stages of selection [22,23]. Spectroscopy-based technologies have been successfully implemented in the last decades to circumvent the stated drawbacks, and are frequently applied by plant breeders and scientists [19,24,25]. The most common technique is near infrared spectroscopy (NIRS), which is based on the emission of near infrared radiation (750–2500 nm) that is absorbed by O-H, C-H, C-O and N-H bonds, the main compounds of plant tissues [19,26], resulting in a unique reflection spectrum for each compound. Therefore, the specific chemical composition of the analyzed material results in a spectral fingerprint [19,26].

A major constraint of NIRS is the missing information about the exact location of individual chemical components inside the sample. This can be resolved by combining spectroscopic and vision techniques, officially termed as hyperspectral imaging (HSI) [27,28]. A hyperspectral image consists of a two-dimensional (classic) image and spectral data as a third dimension. Both are obtained by hyperspectral camera systems creating a so-called three-dimensional data cube [29], which contains the information about the locally different spectral reflectance [27,28]. It should be noted that both NIRS and HSI are much more complex and can only briefly be introduced here (for details about NIRS see Foley et al. [19] and Cen and He [26]; for HSI see ElMasry and Sun [27] and Park and Lu [28]). Both technologies have already been used in a multitude of different fields [30,31], including grain quality analysis [32–34].

However, the spectral data acquisition of NIRS and HSI cannot stand alone, since both need the calibration of models to relate the measured spectra with phenotypic values (e.g. ingredient concentrations or digestibility) [26,27,35,36]. The calibration models are based on a smaller number of samples, which often is a sub-sample of the whole investigated dataset. These samples should ideally reflect the range of variation of the investigated dataset and are analyzed using standard laboratory methods [37]. To a high extent, the quality of the calibration defines the accuracy and precision of predicting the values of the trait of interests by spectral technologies [19,26,27,35,36]. One open question is how to size the calibration dataset to obtain high prediction accuracy while keeping wet chemistry costs low.

The specific objective of the present study was the examination of different calibration model designs and their impact on prediction performance of hyperspectral imaging as high-throughput tool for grain quality analysis using the wild barley population HEB-YIELD [38]. Therefore, we investigated the protein and nutrient concentrations of mature grains via wet chemistry analysis (ICP-OES) and hyperspectral imaging at two European locations in two successive years. The hyperspectral imaging results have been compared to those originating from wet chemistry analysis. Several regression models, calibration set sizes and calibration set compositions have been tested to evaluate the impact of calibration quality on phenotypic value estimation.

## Materials and methods

### Plant material

HEB-YIELD [38], a subset of the wild barley nested association mapping (NAM) population Halle Exotic Barley-25 (HEB-25, [39]), was used in this study. HEB-25 originated from crossing 25 diverse wild barley accessions (*Hordeum vulgare* ssp. *spontaneum* and *H*. *v*. ssp. *agriocrithon*) with the German elite spring barley cultivar Barke (*Hordeum vulgare* ssp. *vulgare*, released in 1996 by breeder Breun). HEB-25 comprises 1,420 BC_1_S_3_ derived lines (backcrossed with Barke), grouped into 25 families (for more details see Maurer et al. [39]).

The HEB-YIELD subset consists of 48 HEB-25 lines that were selected from HEB-25 to ensure good threshability and the absence of brittle rachis, whereby enabling accurate yield estimation in field trials.

### Field trials

The HEB-YIELD population was grown at two locations during two years (2015 and 2016), resulting in four environments. The locations were Dundee (United Kingdom; 56°28'53.71"N 3°6'35.17"W) and Halle (Germany; 51°29'46.05"N 11°59'29.58"E). At both locations the plants were cultivated under regular fertilization and under nitrogen deficiency together with local checks in four replications. Under nitrogen deficiency the lines received no additional mineral N fertilizer. The difference between both treatments regarding N were among 60 and 70 kg/N per hectare in both years by considering the results of the N_min_ analysis, which was performed in early spring prior to sowing to determine the availability of N for the HEB-YIELD lines. A detailed description is given in Wiegmann et al. [40].

The studies were conducted on land owned by the authors' institutions. The research conducted complied with all institutional and national guidelines.

### Phenotypic data

In this study grain elemental concentrations of six agronomically important traits were investigated, including nitrogen (N), phosphorus (P), potassium (K), magnesium (Mg), iron (Fe), and zinc (Zn). A list of these traits is given in S1 Table, including their method of measurement and in which location and year the traits were scored.

In a previous study, based on the same wet chemistry data, it could be shown that the nutrient concentration of grains was not influenced by the conducted N treatment [40]. Therefore, the results of the present paper are based on merged data from both N treatments.

Standard descriptive statistics on raw phenotype data of the investigated traits (see above) were calculated and the coefficient of determination (CV) was defined as $\frac{standard\;deviation}{arithmetic\;mean}$.

### Hyperspectral image recording

Hyperspectral images have been taken in a unique high-throughput phenotyping platform, whose main components are: (1) object plate, (2) white reference, (3) light source, (4) HSI camera and (5) electronically controlled railed carriage (S1 Fig). The phenotypic platform was developed in collaboration with the Fraunhofer Institute for Factory Operation and Automation (IFF).

For achieving a low and homogenous reflection background across the investigated wavelengths the object plate was coated in black fleece. As white reference the Zenith Lite diffuse reflectance target (SphereOptics GmbH, Herrsching, Germany) with a reflection of 95% (spectralon) was used and scanned for each grain sample. The grain samples haven been illuminated through two 150 W quartz halogen lamps in combination with two reflectors to avoid a loss of radiation intensity. These lamps were positioned in a 45° and 135° angle relative to the horizontally placed grains on the object plate. In addition, the image acquisition was conducted in a shaded room without external light sources, except the mentioned halogen lamps and the phenotyping platform was covered with black molleton. The heart of the whole platform was the HySpex SWIR 384 hyperspectral pushbroom camera (HySpex, Skedsmokorset, Norway), which had the capacity to encompass a spectral range of 970 to 2500 nm (near-infrared region) with 288 bands. These bands were equally spaced across the spectral range. The camera was equipped with a lens of 30 cm fixed focal length. Both the HSI camera and the light source were mounted on an electronically moveable railed system with a distance of 30 cm to the grain sample underneath of it. With this setup 16 Bit digitized high resolution reflectance data with 384 spatial pixels in line at a maximal achievable frame rate of 400 Hz were obtained.

The spectral data for the 1,593 grain samples investigated in this study have been obtained through the above described phenotyping platform and all samples were subsequently analyzed via wet chemistry as described in the next chapter.

### Nutrient analysis via wet chemistry

After air drying the harvested grains for two weeks, 6-8 g of grains of each plot were ground and homogenized using the mixer mill MM 400 (Retsch GmbH; Haan, Germany).

The dry matter concentration (DM) of each sample was determined after drying the barley flour for 3 hours in a drying cabinet at 105°C (method 3.1 modified [40]).

The element N was measured with a CNS analyzer (vario EL cube; Elementar Analysensysteme, Langenselbold, Germany), which is based on combustion analysis [40].

For determination of the macronutrients (P, K & Mg) and micronutrients (Fe & Zn) inductively coupled plasma—optical emission spectrometry (ICP-OES) was used (Varian 715-ES ICP-OES; Varian, Palo Alto, California, USA). For more details about wet chemistry analysis, see Wiegmann et al. [40].

### Nutrient analysis via hyperspectral imaging

Hyperspectral image cubes were processed by the automated workflow system HawkSpex Flow developed by the Fraunhofer IFF written in Matlab (Mathworks Inc.). In order to obtain reflectance values, the white target was automatically marked and extracted. Reflectance calculation was performed using

$$R_{\lambda }=\frac{I_{\lambda }-I_{\lambda }^{DC}}{I_{\lambda }^{W}-I_{\lambda }^{DC}}$$

where *I*_*λ*_ is the image pixel intensity at wavelength *λ*, $I_{\lambda }^{DC}$ the intensity when measured with closed shutter (“dark current”) and $I_{\lambda }^{W}$ being the intensity while recording the spectralon device. For a number of images a Neural Gas algorithm [41] was used to cluster the principal material groups in the image (spectralon, table surface, grains). The cluster mask representing the grain material was manually selected and corrected. These segmentation masks defined the identity of foreground (grain) and background (spectralon, table surface) pixels. A Radial Base Function (RBF) Neural Network [42] was then trained as classifier to separate foreground and background. This classifier was then applied to all grain images and yielded a robust and fully automated separation of grains and background.

Pixels representing grain material were then collected and their respective spectrum per grain image was averaged. These average spectra were used as input for a regression model, where a nutrient served as target value. In order to test the effect of different sample sizes, several validation schemes were performed with 5%, 10%, 20%, 40%, 60% or 80% of the target values being randomly included in the calibration set. Sample selection was independent of genotype replications, but stratified for the treatment (1:1). In each validation round, the given percentage of samples was then used to calibrate the regression model while the remaining samples served as test samples. In total, 100 validation rounds with the respective random split were calculated. Additionally, a leave-one-out scheme was used where in each validation round one sample is left out of the training set (= N-1; for simplicity referred to as 99%). In this scheme, the number of samples in a particular set determines the number of validation rounds in the modelling. In the leave-on-out scheme, no random sample drawing is performed.

As performance measure for prediction, the coefficient of determination (R^2^) was used. R^2^ was defined as the squared Pearson correlation coefficient:

$$R^{2}={\left(\frac{\sum_{i=1}^{n}\left(y_{i}-\bar{y})(t_{i}-\bar{t}\right)}{\hat{\sigma_{y}}\hat{\sigma_{t}}}\right)}^{2}$$

where *y*_*i*_ is the nutrient prediction for sample i, while *t*_*i*_ is the target (true) nutrient value with $\bar{y}$ and $\bar{t}$ being their respective averages as well as $\hat{\sigma_{y}}$ and $\hat{\sigma_{t}}$ being their respective standard deviations. A perfect prediction is achieved with an R^2^ of 1.0. The threshold of R^2^ values, above which a sufficient prediction is achieved, is debatable.

As regression models, a Partial Least Squares (PLS) Regression Model, which is a basic method in optical chemometrics [43], along with two neural network types, a Radial Base Function with Transfer Learning (tRBF) Neural Network [44] and a Multi-Layer Perceptron Network [45] were applied (for more details see Table 1).

**Table 1:** Regression model details.

Model	Hyperparameters	Learning Rule
Partial Least Squares (PLS)	PLS Components = 20	Method of the smallest squares
Radial Base Function Network with Transfer Learning (tRBF)	Radial Basis Function = 20 Metric = Euclidean Distance	Scaled Non-Linear Conjugate Gradient; Matlab Package minFunc
Multi-layer Perceptron (MLP)	Two Hidden Layers Hidden Layer 1 = 30 Neurons Hidden Layer 2 = 10 Neurons Hidden Layer Activation = tansig Output Layer Activation = linear	Levenberg–Marquardt; Matlab Neural Network Toolbox

A PLS model finds a linear regression model by projecting the predicted variables and the observable variables to a new space similar to a principal component analysis (PCA). In contrast to a PCA, PLS is finding hyperplanes of maximum variance between the response or target value and independent or observed variables. PLS model parameters are found by least squares method. The number of PLS components was manually set to 20.

Data-driven learning methods like Artificial Neural Networks (tRBF and MLP) try to model a system behavior not by formulating a physical model but parameterizing a general purpose numerical structure. In general, an Artificial Neural Network derives its idea from the information and learning process in the human brain, where a large number of simple processing units are linked together by weighted connections. Technically, a neural network is a universal function approximation system. A numerical model generates an output from an input via structure neurons. The output is compared to a target value (or ground truth value) and an error value is calculated, the so-called loss function. The learning parameters then adjust the weighted connections of the network iteratively so that the error produced by all training samples is minimal. In that way, a generic numeric function is fitted to an input/output problem and generates in our case a regression model for predicting nutrient concentration (output) from spectral reflectance measurements (input) without the need to model a physical process how a reflectance is produced by a nutrient concentration. The parameters of the applied tRBF and MLP neural networks are found by numerically optimizing the objective function of mean squared error (MSE) between target and prediction value. Optimization is performed using a gradient descend approach and stopped if a number of epoch (1000) is reached or the MSE converges, e.g. changes in MSE fall below a defined threshold of 1e-05.

The tRBF models the dataspace as a weighted mixture of Gaussian kernel functions calculated via distance calculation of the input sample towards prototypical patterns retained in the model, while MLP tries to model the data via the use of hyperplanes.

Calibrating a number of different regression models is a typical approach in machine learning since it is difficult to assess the nature of a high-dimensional dataspace and to decide whether the systematic relationship between the spectrum and the nutrient is linear (PLS) or non-linear (tRBF, MLP).

Modelling was performed on separate datasets for single environments, as well as for a two-year model per location and across all four environments. In order to test the transferability of the models, samples that were not used for model training were predicted and the prediction quality was assessed with the R^2^ measurement as described above.

### Cost benefit analysis

In order to estimate the relative prediction performance gain with increasing sample number, a cost benefit analysis was carried out between two consecutive calibration set sizes, each based on the following formula,

$$\frac{\Delta prediction\;performance}{\Delta sample\;number}$$

with Δ indicating the difference between two consecutive calibration set sizes with regard to prediction performance (e.g. R^2^_10%_—R^2^_5%_) and sample number (e.g. N_10%_—N_5%_), respectively.

### Statistical analyses

SAS 9.4 (SAS Institute Inc., Cary, NC, USA; [46]) was used to estimate variance components for each environment separately with *PROC VARCOMP* by including the random factor genotype to explain a trait. Based on the estimated variance components repeatabilities (rep) were calculated within each environment:

$$rep=\frac{V_{g}}{V_{g}+\frac{V_{r}}{R}}$$,

where

*V*_*g*_ = genotype variance (based on 48 genotypes)

*V*_*r*_ = residual variance

*R* = number of replicates (4)

The different regression models and calibration set compositions have been investigated for statistical significance regarding their prediction performance through the results of a one-factorial (factors regression model and calibration set composition, respectively) ANOVA (R package “stats” 3.6.1) and a subsequent Tukey’s test ([47]; R package “agricolae” 1.3.1). A Fisher’s z transformation ([48]; R package “psych” 1.8.12) was applied over Pearson’s correlation coefficients of prediction performance to account for non-normal distribution. We checked for homogeneity of phenotypic variances between the random sampling of the three regression models (PLS, MLP, tRBF) to rule out that differences in prediction performance between them were caused by differences in phenotypic variances by applying Fligner-Killeen tests ([49]; R package “stats” 3.6.1).

All figures were created using R 3.6.1 [50] with the package “ggplot2” 3.2.0 [51], except S14 Fig, which was created with SAS *PROC SGPANEL*.

## Results and discussion

### Phenotypic data

Every spectral-based technology depends on measuring a subset of the samples via wet chemistry analysis to generate a calibration model to link the spectra with the phenotypic values determined in the laboratory [27,35,36,52]. In the present study the full set of all 1,593 samples from the wild barley introgression population HEB-YIELD, grown in Dundee (United Kingdom) and Halle (Germany) in 2015 and 2016, has been measured using wet chemistry to determine six grain nutrients, including four macronutrients (N, P, K & Mg) and two micronutrients (Fe & Zn) (S2 Table). The majority of these traits showed a considerable amount of variation indicated by the coefficient of variation (CV), which ranged from around 6% for Mg in Halle 2015 to more than 23% for Fe in Dundee 2016 (S2 Table). Moreover, the average repeatability of 0.93 for the six nutrient traits indicates that the effect of the genotype on these traits is high and the residual variance is comparatively low, also hinting on trustworthy wet chemistry measurements (S2 Table).

Prior to the wet chemistry analysis, the hyperspectral reflectance of each grain sample has been captured via HSI by using the same grains that were utilized for subsequent wet chemistry analysis. Finally, all 1,593 samples were analyzed via wet chemistry (S3 Table) and hyperspectral imaging to determine grain nutrients.

The resulting dataset was used in a case study to investigate the impact of different calibration models on prediction performance of hyperspectral imaging for nutrients in mature barley grains. The calibration models varied based on the applied regression model, the number of samples used for the calibration set, as well as the sample selection for the calibration sets, which was either conducted within a single environment, across years, or across environments. The coefficient of determination (R^2^) serves as measure for the prediction performance of the calibration models throughout the study.

### Comparison of regression models

Independent of the material (e.g. grains, food or landscapes) that is scanned by a HSI camera system, the resulting spectra need to be linked to a target trait (e.g. phosphorus content, free fatty acids or soil type) by applying an adequate regression model [27,35,36]. Three regression models, based on multi-layer perceptron (MLP), radial base function network with transfer learning (tRBF) and partial least squares (PLS), were tested to evaluate if the model type affects prediction performance of grain nutrients.

In accordance to a multitude of spectral-based studies originating from various fields of research [53–57], the choice of a suitable calibration model is also critical for predicting grain nutrients.

**Fig 1:**
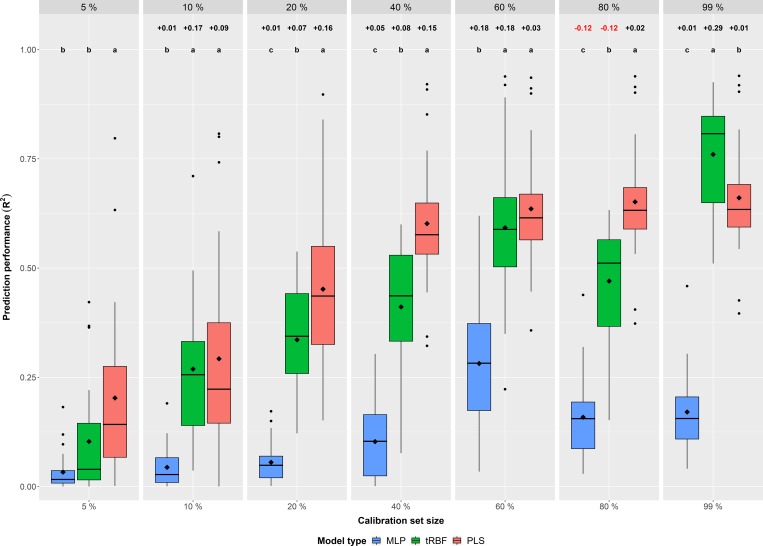
Regression model comparison—Across environments—Across traits. Comparison of the investigated regression models in regard to prediction performance (R^2^) across the four environments (DUN15, DUN16, HAL15 & HAL16) and the six nutrient traits (N, P, K, Mg, Fe & Zn) for different calibration set sizes from 5% to 99%. The color of the boxplots differentiates the three different model types MLP (multi-layer perceptron, blue), tRBF (radial base function network with transfer learning, green) and PLS (partial least squares, red). The diamonds inside the boxes indicate the arithmetic mean. Letters (a, b, c) in the upper part of the figure indicate significant (P<0.05) differences between the models based on a Tukey test (S4 Table). Furthermore, numbers above the letters indicate the change in prediction performance compared to the next smaller one.

The combined data of the four environments, averaged across all six nutrients, revealed a clear ranking of the regression models, where the best predictions were achieved with PLS followed by tRBF and MLP (Fig 1). This trend was also valid by looking at the results for single environments (S2 Fig; S3 Fig; S4 Fig; S5 Fig) and single nutrients (S6 Fig). A Tukey test confirmed the low performance of the MLP model, since its predictions were significantly below the average prediction performances of the two remaining models (S4 Table). The predictions made with the tRBF model were in all calibration set sizes, except the largest one (99%), below the average of PLS, although statistically not always significant (S4 Table).

Furthermore, the regression models can be differentiated based on their computing demand, which increases in the following order: PLS < tRBF < MLP (on average 0.2 s < 20 s < 50 s per single model in our dataset). It should be noted that the computing demand to generate the calibration models is substantial, even if high computing performance systems are available. Therefore, it represents an additional factor in choosing an adequate model.

Due to the good prediction performances of the PLS model and the lowest computing demand all following results are exclusively based on PLS (results of MLP and tRBF are available in Supplementary Tables). The PLS model is the basic model in optical chemometrics [43] and a well-suited tool for the analysis of spectral data [58,59]. It has been successfully applied in various fields of spectroscopy [60–62]. However, one should note that the suitability of certain regression models is highly dependent on the dataset for the task at hand and an approach of testing different regression methodologies should be followed. In this context it should also be noted that if larger wet lab datasets were available machine learning methods like MLP and tRBF will most likely benefit, giving the possibility of reaching higher predictive abilities.

### Comparison of calibration set sizes

**Fig 2:**
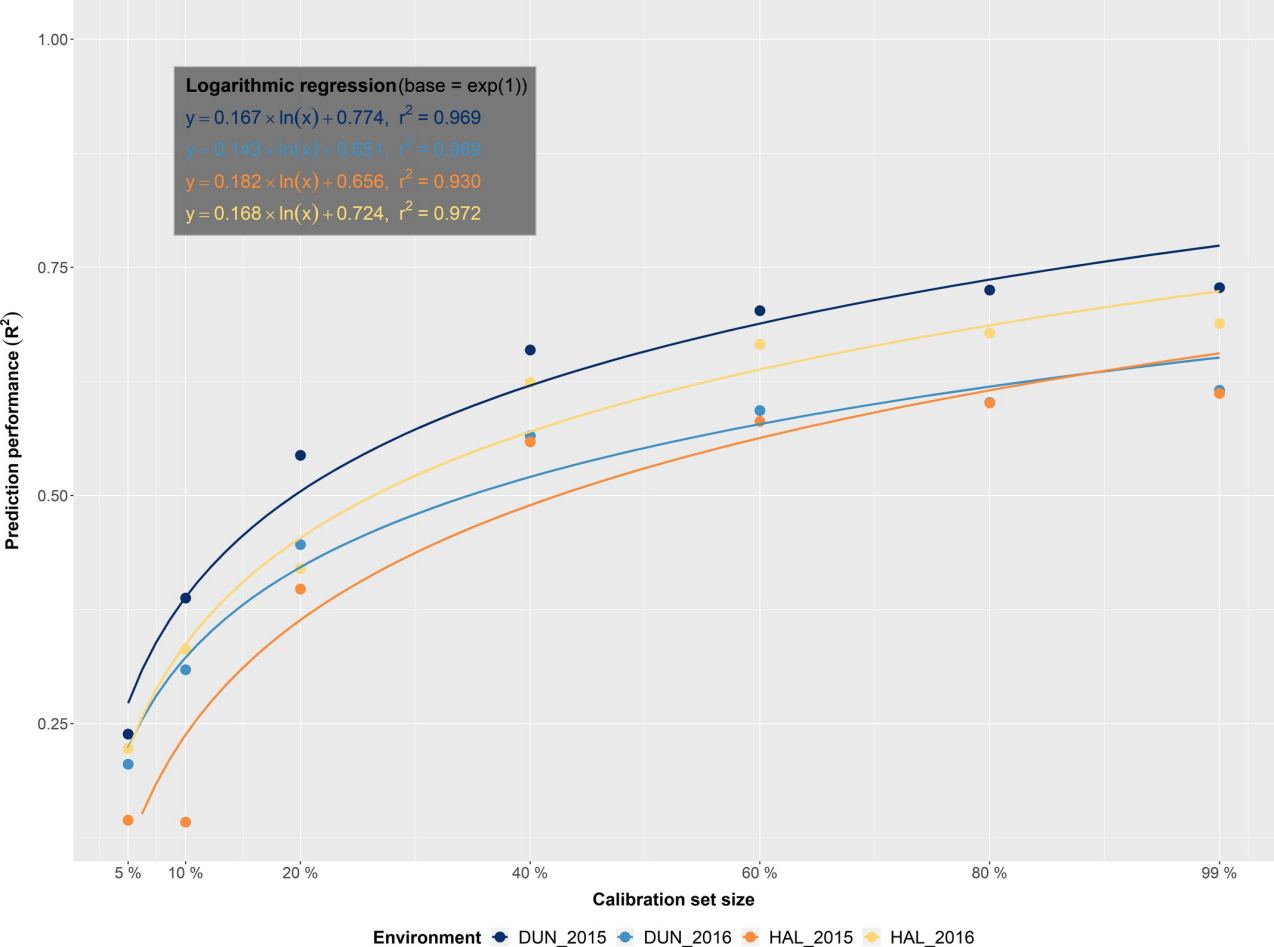
Calibration set size comparison—Within environments—Across traits. Impact of calibration set size on prediction performance (R^2^) in each of the four environments (DUN15 = dark blue, DUN16 = light blue, HAL15 = orange, HAL16 = yellow) across the six nutrient traits (N, P, K, Mg, Fe & Zn). A logarithmic function was fitted, which indicates the gain in prediction performance (R^2^) with increasing calibration set sizes. The formulas of these four functions are shown in the upper left corner.

In the present study all samples were entirely analyzed via wet chemistry, which enabled to flexibly adjust calibration set sizes to find the minimal size for achieving good predictions. As already indicated in Fig 1, the size of a calibration set affects the quality of the calibration model and, finally, the prediction performance of HSI. If money and time would not be limiting factors the best way to obtain trustworthy grain ingredient data would certainly be the analysis of all samples by standard laboratory methods [19–21]. In reality, however, an ideal calibration set has to be defined based on a cost-benefit analysis. On the one side a calibration set needs to be large enough to enable reliable predictions, on the other hand it should not be larger than necessary to avoid excessive wet chemistry costs. Esteve Agelet and Hurburgh [52] indicated that the choice of the right calibration set is frequently underestimated, even though it defines the quality of spectroscopy-based analyses. Therefore, we created individual calibration models with seven different sample sizes (5% 10%, 20%, 40%, 60%, 80% and 99%, reflecting an approximate sample number of n≈20, 40, 80, 160, 240, 320 and 400 in each environment, respectively) for the six nutrient traits. On average, in each environment an enhancement of the calibration set resulted in an improvement of the prediction performance. This increase can be described through a regression based on the natural logarithm in all four environments (mean R^2^ of 0.96; Fig 2; S5 Table).

**Fig 3:**
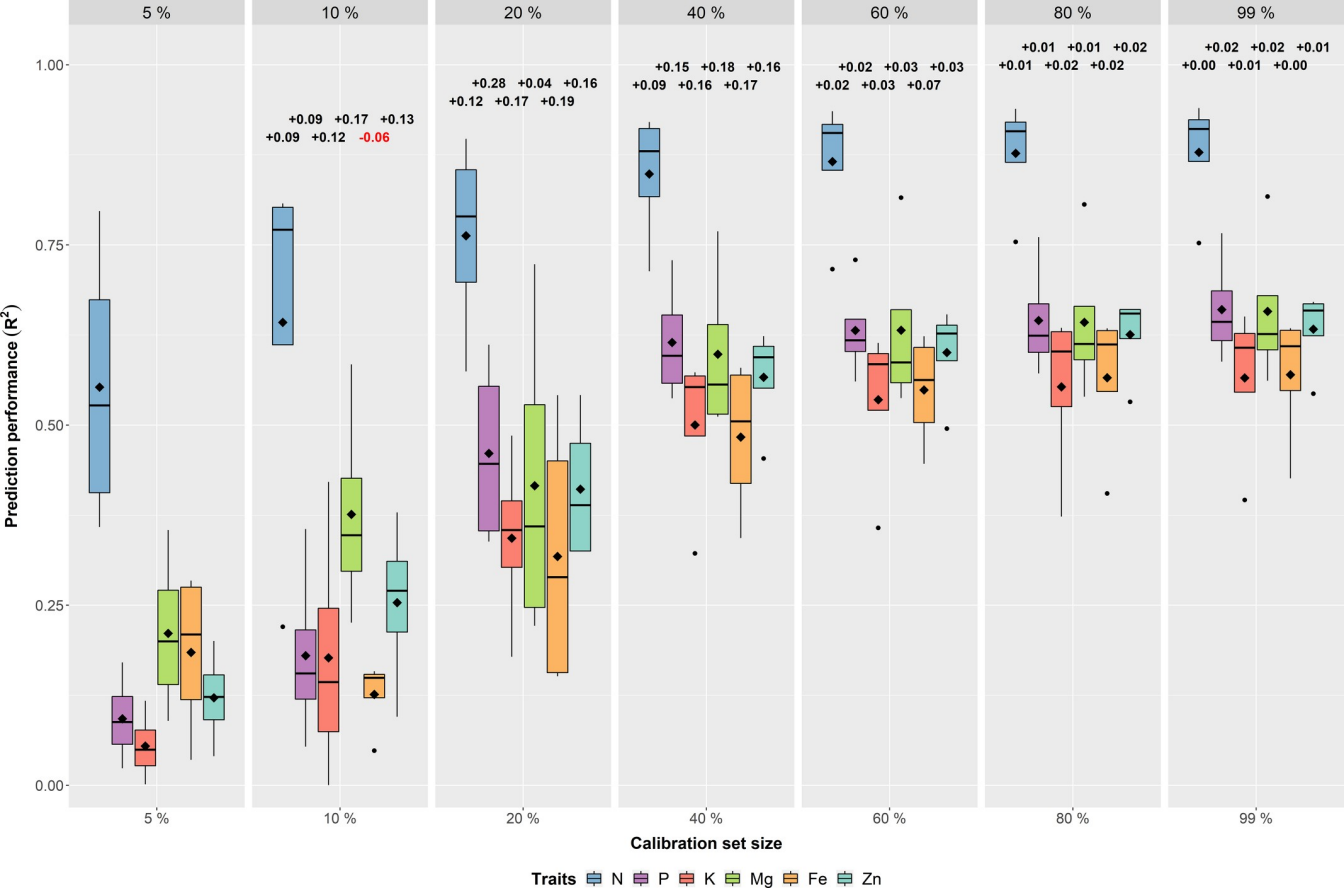
Calibration set size comparison—Across environments—Within traits. Impact of calibration set size on prediction performance (R^2^) across the four environments (DUN15, DUN16, HAL15 & HAL16) for each of the six nutrient traits (N, P, K, Mg, Fe & Zn). The color of the boxplots represents the six different traits and the diamonds inside the boxes indicate the arithmetic mean. The numbers in the upper part of the figure indicate the change in prediction performance compared to the next smaller one.

The effect of the calibration set size has also been investigated for each nutrient across the four environments (Fig 3; S5 Table), as well as within each of them separately (S5 Table; S7 Fig; S8 Fig; S9 Fig; S10 Fig). For all nutrients the same trends regarding the calibration set size effect on prediction performance could be observed. By far the best values could be obtained for N, reflecting the grain raw protein content, which reached R^2^ values >0.9. For this nutrient, a calibration set of 40 samples (10%) was sufficient to achieve reliable measurements with an average R^2^ of 0.65. The good predictions for N are in agreement with trustworthy prediction of N by using NIRS [35,63,64]. For instance, Velacso and Möllers [63] found an R^2^ of 0.94 between NIRS and combustion analysis for protein content in rapeseed. The nutrients P, K, Mg, Fe and Zn were characterized by intermediate prediction performances, indicated by mean R^2^ values of >0.48 at a calibration set size of n = 160 (40%).

The effect of the calibration set size on prediction performance was different for each trait. However, a general pattern existed that appreciable improvements were possible until a calibration set size of 160 samples (40%) was reached (Fig 3; S11 Fig; S12 Fig; S13 Fig). From this stage on a plateau was reached and each further added sample could only marginally increase R^2^ by ≈0.0004 (S6 Table). This finding may be explained by the fact that the variation of the samples in the calibration set at this stage already adequately reflects the variation of the whole dataset, which is one requirement for valid predictions [37,52]. With increasing calibration set size the range of covered trait values also increases, which might lead to a better predictive model. The high mean correlation coefficient of 0.93 between the trait value range covered by the calibration set and the prediction performance (R^2^) confirms this assumption (S14 Fig).

By looking at the impact of calibration set size on prediction performance in each environment individually (S7 Fig; S8 Fig; S9 Fig; S10 Fig; S11 Fig; S12 Fig; S13 Fig), it is frequently observable that the performance fluctuates in smaller calibration sets (5%, 10% and 20%). This is especially pronounced in Halle 2015 for the 10% calibration set size, which gives worse predictions than the 5% calibration set size (S9 Fig). We also observed this in the remaining environments like in Dundee 2015 for Fe (S7 Fig), in Dundee 2016 for K and Fe (S8 Fig) and in Halle 2016 for N, P and Mg (S10 Fig). This observation is unexpected, since in general larger calibration sets should lead to more trustworthy predictions [65]. It may be explained by the fact that in small calibration sets the probability is higher that by chance the selected samples do not adequately reflect the variation of the investigated population. The importance of having representative samples in a calibration set is well-known and has already been investigated decades ago [37,66–68]. Also overfitting might play a role in this context, which was observed in small calibration set sizes (0.05 and 0.1), indicating that results gathered from these calibration set sizes should be taken with caution (S5 Table).

However, the general trend that higher calibration set sizes positively influence prediction performance is undisputable and based on the results the recommended calibration set size should be around 160 samples to achieve reliable predictions with an R^2^ of 0.5 for P, K, Mg, Fe and Zn, whereas for N already 80 samples are adequate. It should be stated that most measurements related to plant breeding are affected by population-specific effects [69–71], which will also apply to the HSI analysis of grain ingredients. Therefore, the presented results should always be evaluated against the background of the examined wild barley population HEB-YIELD.

### Expanding calibration set models

It is well-known that different years and locations impact plant characteristics like height or grain yield [69,72,73], which also holds true for the concentration of nutrients in mature grains in barley [40]. Therefore, calibration models should be recurrently upgraded to increase their flexibility [33,37,68]. The studies of León et al. [74] and Roger et al. [75], conducted in olive fruits and wheat grains, respectively, support the negative impacts of uncontrollable effects (e.g. year) on prediction performance, which can be alleviated by expanding the calibration models through the inclusion of samples from several years.

**Fig 4:**
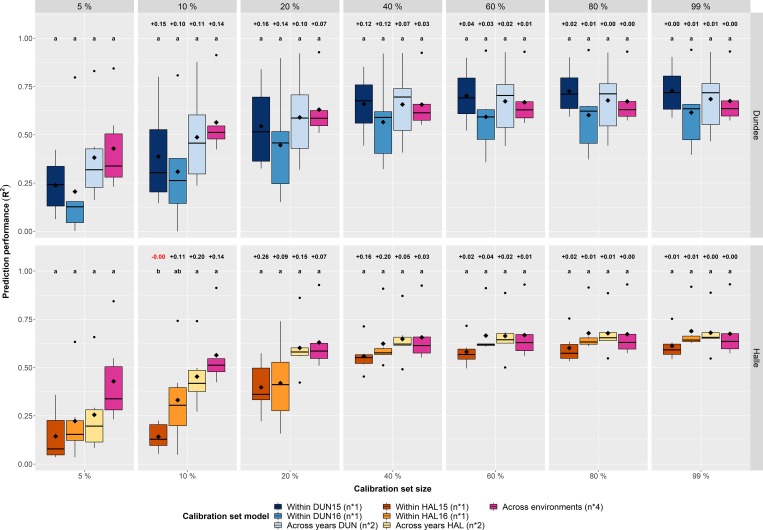
Calibration model comparison—With additional samples—Within environments—Across traits. Comparison of the three calibration set compositions (within environments, across years & across environments) across the six nutrient traits (N, P, K, Mg, Fe & Zn) in Dundee and Halle. The color of the boxplots represents the combination of the different calibration set models and environments. The resulting extension of the total number of samples used for the respective model composition is indicated in parentheses (n*1 = single number of samples, n*2 = duplicated number of samples & n*4 = quadruplicated number of samples). The diamonds inside the boxes indicate the arithmetic mean. Letters (a, b) in the upper part of the figure indicate significant (P<0.05) differences between the model compositions based on a Tukey test (S7 Table). Furthermore, numbers above the letters indicate the change in prediction performance compared to the next smaller one.

**Fig 5:**
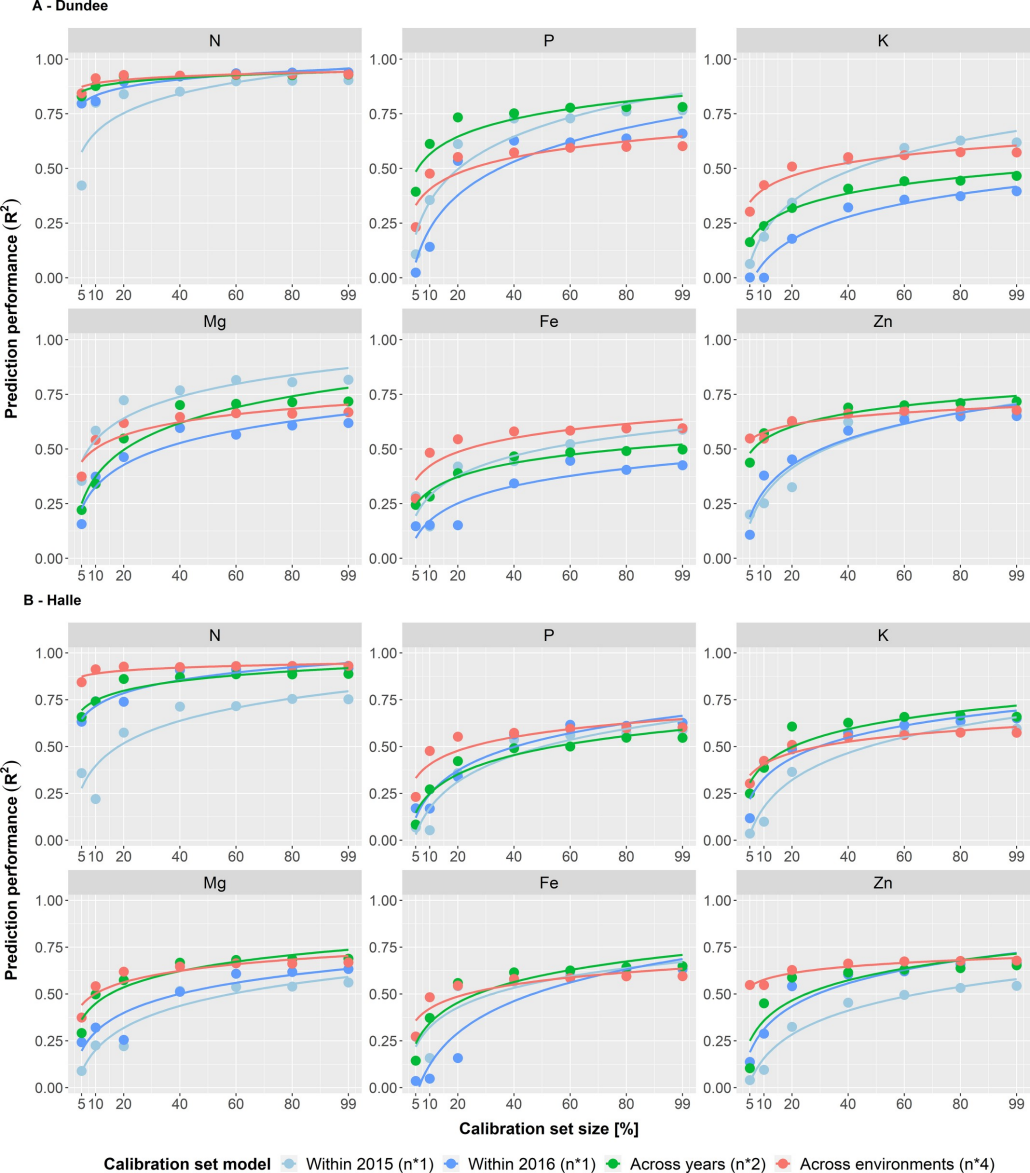
Calibration model comparison—With additional samples—Within environments—Within traits. Comparison of the three calibration set compositions (within environments, across years & across environments) for each of the six nutrient traits (N, P, K, Mg, Fe & Zn) in Dundee and Halle. The colors of the lines represent the different calibration set models. In addition, the legend contains the number of samples used for the respective model composition (n*1 = single number of samples, n*2 = duplicated number of samples & n*4 = quadruplicated number of samples) in parentheses.

Therefore, the calibration models have been expanded by duplicating (across years) or even quadruplicating (across environments) the sample number of the calibration sets by using equal sample numbers from each year or each environment. For instance, if in the single environment approach 80 samples were used, 160 were used for the across years and 320 for the across environments approach, respectively. This resembles the common procedure in NIRS where the calibration models are expanded successively by including data from several years and locations [52,76–78]. Both the across years and the across environments approach clearly improved the predictions of grain nutrients, especially in calibration sets with a lower sample size (Fig 4; S5 Table; S7 Table). Furthermore, both approaches clearly reduced the variance of the predictions, as indicated by a lower range as well as smaller coefficients of variation for sample sizes <160 (S8 Table). By looking at the second smallest calibration set (n = 40) in Halle, the average R^2^ was 0.14 in 2015, whereas the mean R^2^ was increased to 0.45 and 0.56 when predicting based on the across years approach and the across environments approach, respectively (S8 Table). The extension of the calibration model with data of two years could triplicate the average prediction performance in comparison to the single environment approach Halle 2015, while the across years approach contained 80 samples versus 40 samples in the single environment approach. However, further extension of the model with data from two locations revealed only a smaller increase to 0.56 at a calibration set size of 160. The across environments approach reached its maximum prediction performance in the calibration set containing 40% (n = 640) of the samples with an average R^2^ of 0.66. Further sample enhancements hardly impacted prediction, which might be the consequence of little additional variation from the additional samples. Only few nutrients showed better predictions in small calibration set sizes with the single environment models (Fig 5; S5 Table). The results confirm the advantage of adding samples from additional environments to calibration models to improve prediction performance as commonly done in NIRS [52,76–78]. Finally, it should be stated that the generation of such complex calibration models is time-consuming (up to several years) and expensive since a higher number of samples from several environments needs to be analyzed by means of wet chemistry.

### Transferability of models

Since model implementation is complex, especially when upgrading it successively, a desirable approach would be to develop only a single robust model, which could be transferred to all kinds of environments without additional efforts (also known as external calibration). The idea of transferring models or keeping them robust over longer times is not new [79] and has been investigated in spectroscopic studies with diverse backgrounds [26,74,80], since it would enable to circumvent the obstacles stated above.

**Fig 6:**
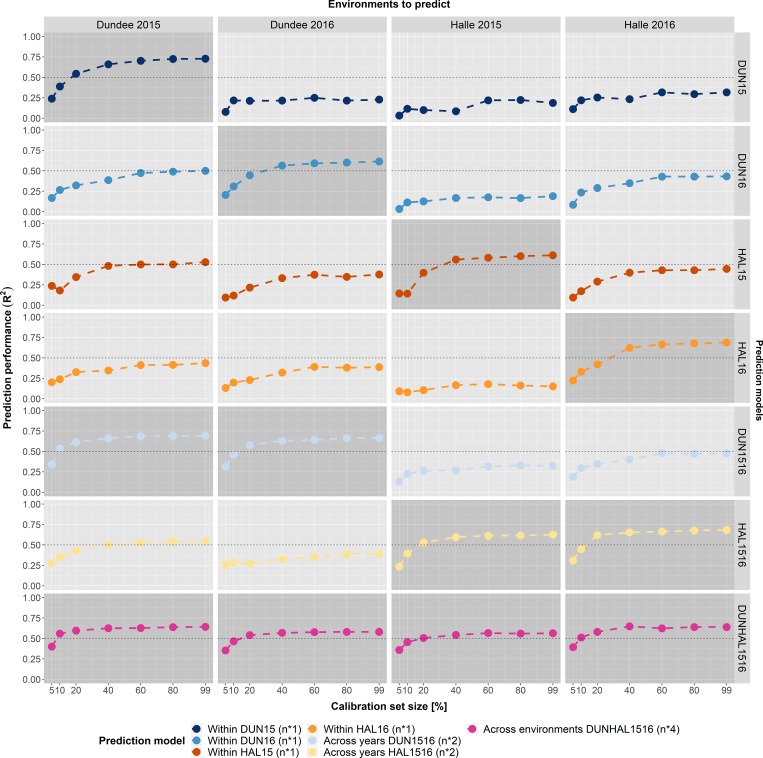
Model transferability—Within environments—Across traits. Evaluation of model transferability to predict grain nutrients in each of the four environments (Dundee 2015, Dundee 2016, Halle 2015 & Halle 2016, shown as columns) across the six nutrient traits (N, P, K, Mg, Fe & Zn). Seven different prediction models (within each environment, across years, across environments; shown as rows) were used to predict nutrient concentrations of the six traits in the four investigated environments. Prediction models containing the respective environment to be predicted are visually emphasized. The three types of prediction model compositions contain different numbers of samples: the four within environment models (DUN15, DUN16, HAL15 & HAL16) contain the simple number of samples of the respective environment, the two across years models (DUN1516 & HAL1516) the duplicated number of samples and the across environments model (DUNHAL1516) the quadruplicated number of samples.

Therefore, we investigated how far our developed models are able to predict each single environment. In a first step each single environment model (e.g. Halle 2015; HAL15) was used to predict the four environments (Dundee 2015, Dundee 2016, Halle 2015 & Halle 2016) to obtain an idea of model transferability. As a result, none of the single environment models could reliably predict another environment except its own (Fig 6; S9 Table). The single environment models never reached R^2^ values above 0.5, averaged across the traits, in the non-trained environments. This observation also holds true for each single nutrient, except for N (S9 Table; S15 Fig). It is well-known that N is a reliably predictable nutrient [35,63,64], which is in agreement to the present results where the predictions for N reached R^2^ values above 0.5 in the non-trained environments, even in calibration sets with only 10% of the maximum number of samples. However, it should be stated that the predictions considerably varied between calibration set sizes. By expanding the prediction models with samples from a second year (e.g. DUN15 and DUN16 = DUN1516) they were able to predict both years, but still failed to estimate the nutrient concentrations in both years of the other location. The next logical step was to incorporate data from all four environments into one model (DUNHAL1516) and to use this model to predict the nutrient concentrations in the four environments. The outcome was a full model that contains data from all investigated environments that is able to predict the nutrients in a reasonable order in all environments. Interestingly, the four within environment approaches still outperformed the joint model in their own trained environment, though only at higher calibration set sizes.

A transfer of models in the current scope of this study seems difficult. Since only two years and two locations are available, the probability is high that due to variations between environments and years, the model performance is weakened. For a more robust model, more years and locations should be considered to increase the probability that similar environments are learnt with the calibration dataset. Other studies already pinpointed the expected complexity of a purely data driven approach [26,79,81]. Moreover, as we only investigated one single highly diverse population, we cannot answer the question whether the results also hold true for other less diverse populations and whether trans-populational prediction would be possible.

Finally, a suggestion for users should be to analyze a relatively small number of samples in each location over several years to keep the cost for wet chemistry as low as possible while benefitting from the additional variation introduced through different locations and years into the calibration model. The presented results indicate that the across environments approach outperforms models within a single environment, especially if the sample number of calibration models is low (Fig 4). However, the quality of HSI predictions is excelled by classical laboratory methods [40], which might be acceptable in specific situations. For instance, modern breeding programs consist of thousands of individual genotypes, especially in early generations, where frequently a negative selection is applied to separate the wheat from the chaff. The superior speed of HSI allows breeders to obtain quality-related data already in those early generations, which would be unaffordable with wet chemistry methods.

## Conclusions

Hyperspectral imaging offers users the possibility to analyze their samples in high throughput for a wide range of issues like soil composition and food safety [28,82]. Nevertheless, every spectral-based technology measures only a unique spectrum of a sample to correlate it to the investigated trait (e.g. protein content) based on a calibration model. The importance of these models is frequently underestimated as mentioned by Esteve Agelet and Hurburgh [52]. In the present study we evaluated different model design parameters and could provide information about the optimal model design, exemplified for nutrient content in mature barley grains.

In the dataset presented in this study, a linear regression model based on partial least squares (PLS, [43]) outperformed complex models based on neural networks, since it offered the best prediction performance while minimizing computational demand. Furthermore, we observed a positive relationship (mean R^2^ of 0.96 in a logarithmic regression) between calibration set size and prediction performance with a local optimum at a calibration set size of 160 samples, representing 40% of the data investigated in this study. Above this point further increments in calibration set size are dispensable, since they seem to add no more variability to the calibration model. Models obtained in a certain environment were only to a limited extent transferable to other environments, considering the scope of this study. Extending those models with additional samples from other environments considerably improved the calibration performance. Models should be successively upgraded with new calibration data to enable a reliable prediction of the desired traits in future studies and practical applications of hyperspectral imaging systems, for instance in future plant breeding concepts. Furthermore, model transfer strategies should be investigated to transfer models to unknown environments.

## Supporting information

S1 Table List of scored traits. (XLSX)

S2 Table Descriptive statistics—Wet chemistry. (XLSX)

S3 Table Raw data. (XLSX)

S4 Table ANOVA—Regression model comparison. (XLSX)

S5 Table Correlations and R^2^. (XLSX)

S6 Table Cost benefit analysis—Additional samples—Delta. (XLSX)

S7 Table Calibration model comparison—ANOVA & Tukey. (XLSX)

S8 Table Descriptive statistics—HSI. (XLSX)

S9 Table Model transferability R^2^. (XLSX)

S1 Fig Hyperspectral imaging laboratory rack. (PDF)

S2 Fig Regression model comparison—Dundee 2015—Across traits. (PDF)

S3 Fig Regression model comparison—Dundee 2016—Across traits. (PDF)

S4 Fig Regression model comparison—Halle 2015—Across traits. (PDF)

S5 Fig Regression model comparison—Halle 2016—Across traits. (PDF)

S6 Fig Regression model comparison—Across environments—Within traits. (PDF)

S7 Fig Calibration set size comparison—Dundee 2015—Within traits. (PDF)

S8 Fig Calibration set size comparison—Dundee 2016—Within traits. (PDF)

S9 Fig Calibration set size comparison—Halle 2015—Within traits. (PDF)

S10 Fig Calibration set size comparison—Halle 2016—Within traits. (PDF)

S11 Fig Cost benefit analysis—With additional samples—Within environments—Within traits. (PDF)

S12 Fig Cost benefit analysis—With additional samples—Within environments—Across traits. (PDF)

S13 Fig Cost benefit analysis—With additional samples—Across environments—Within traits. (PDF)

S14 Fig Relationship between trait value range covered by the calibration set and prediction performance (R^2^)—Across environments—Within traits. (PDF)

S15 Fig Model transferability—Within environments—Within traits. (PDF)
