# Supplementary material for: Optimizing the procedure of grain nutrient predictions in barley via hyperspectral imaging
Source: PLoS One. 2019 Nov 7;14(11):e0224491. doi: 10.1371/journal.pone.0224491 (PMC6837513; doi:10.1371/journal.pone.0224491)
Supplement: S1 Fig — (PDF) [file pone.0224491.s010.pdf]

S1 Figure. Hyperspectral imaging laboratory rack

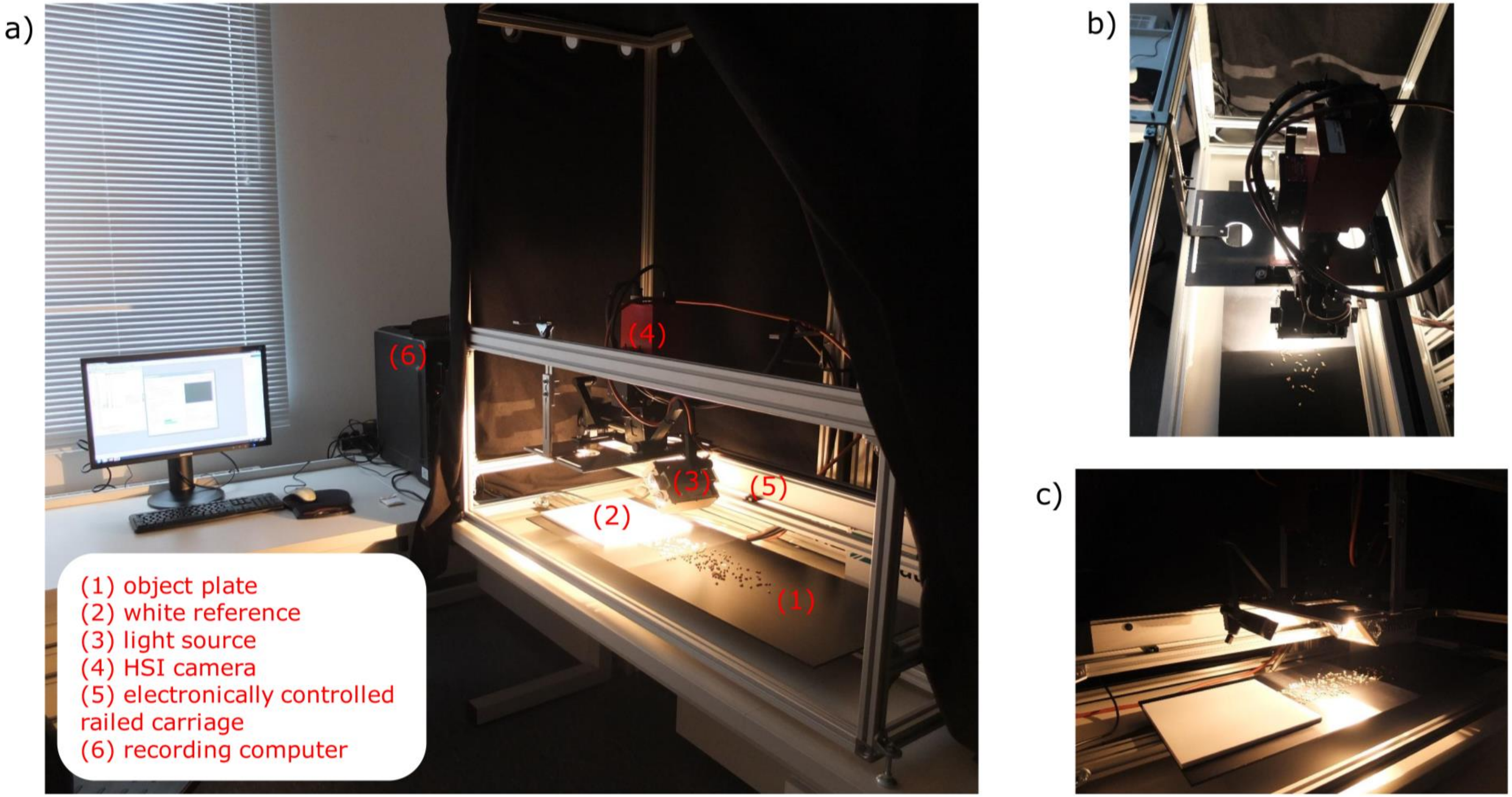

S1 Figure. Setup of the hyperspectral imaging laboratory rack. **a)** overview shot, **b)** top view shot from above the HSI camera and **c)** a close-up view of the object plate. The most important components of the laboratory rack are listed in a). The coating of the object plate with black fleece is not displayed in the present pictures.
