# Supplementary material for: Optimizing the procedure of grain nutrient predictions in barley via hyperspectral imaging
Source: PLoS One. 2019 Nov 7;14(11):e0224491. doi: 10.1371/journal.pone.0224491 (PMC6837513; doi:10.1371/journal.pone.0224491)
Supplement: S2 Fig — (PDF) [file pone.0224491.s011.pdf]

S2 Figure.
Regression model comparison - Dundee 2015 - Across traits

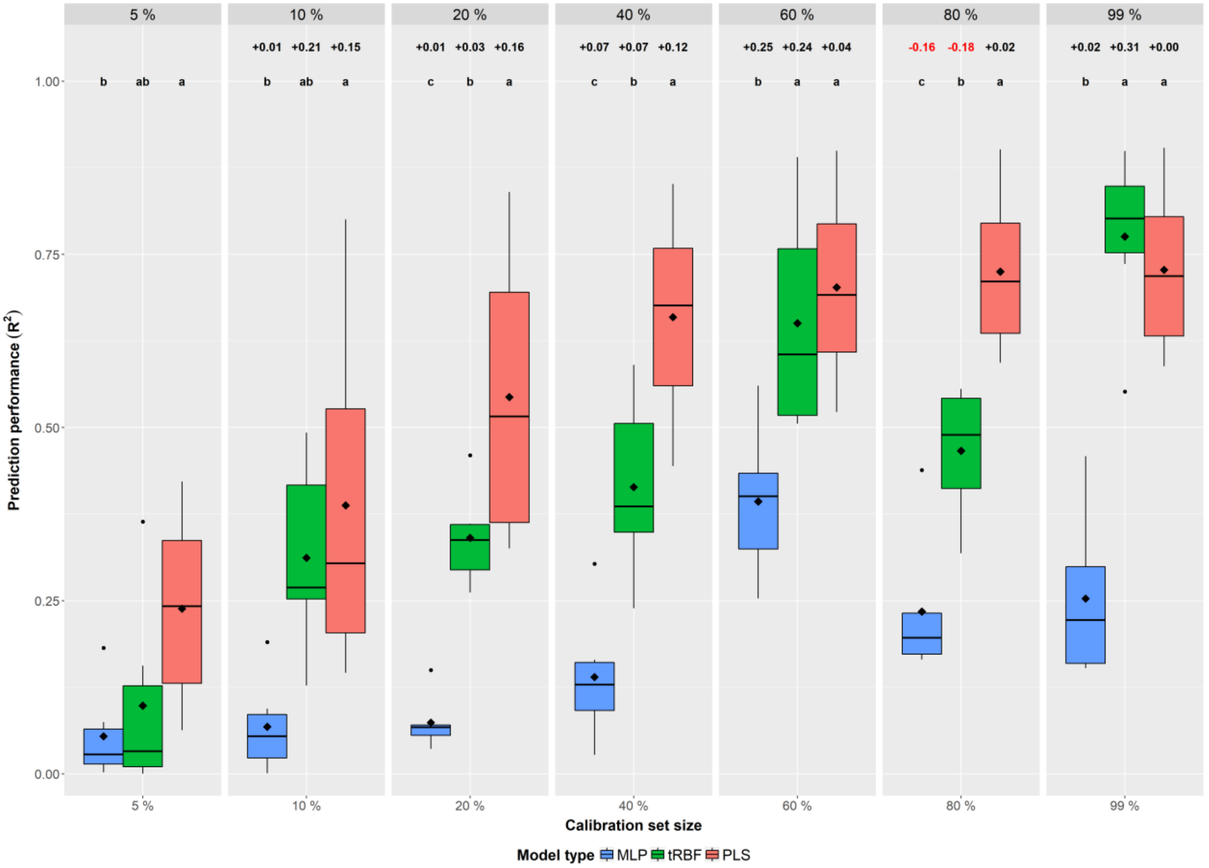

S2 Figure. Comparison of the investigated regression models in regard to prediction performance (R^2) in Dundee 2015 (DUN15) across the six nutrient traits (N, P, K, Mg, Fe & Zn) for different calibration set sizes from 5% to 99%. The color of the boxplots differentiates the three different model types MLP (multi-layer perceptron, blue), tRBF (relevance radial basis function network, green) and PLS (partial least squares, red). The diamonds inside the boxes indicate the arithmetic mean. Letters (a, b, c) in the upper part of the figure indicate significant ($P<0.05$) differences between the models based on a Tukey test. Furthermore, numbers above the letters indicate the change in prediction performance compared to the next smaller one.
