# Supplementary material for: Optimizing the procedure of grain nutrient predictions in barley via hyperspectral imaging
Source: PLoS One. 2019 Nov 7;14(11):e0224491. doi: 10.1371/journal.pone.0224491 (PMC6837513; doi:10.1371/journal.pone.0224491)
Supplement: S6 Fig — (PDF) [file pone.0224491.s015.pdf]

S6 Figure. Regression model comparison -Across environments -Within traits

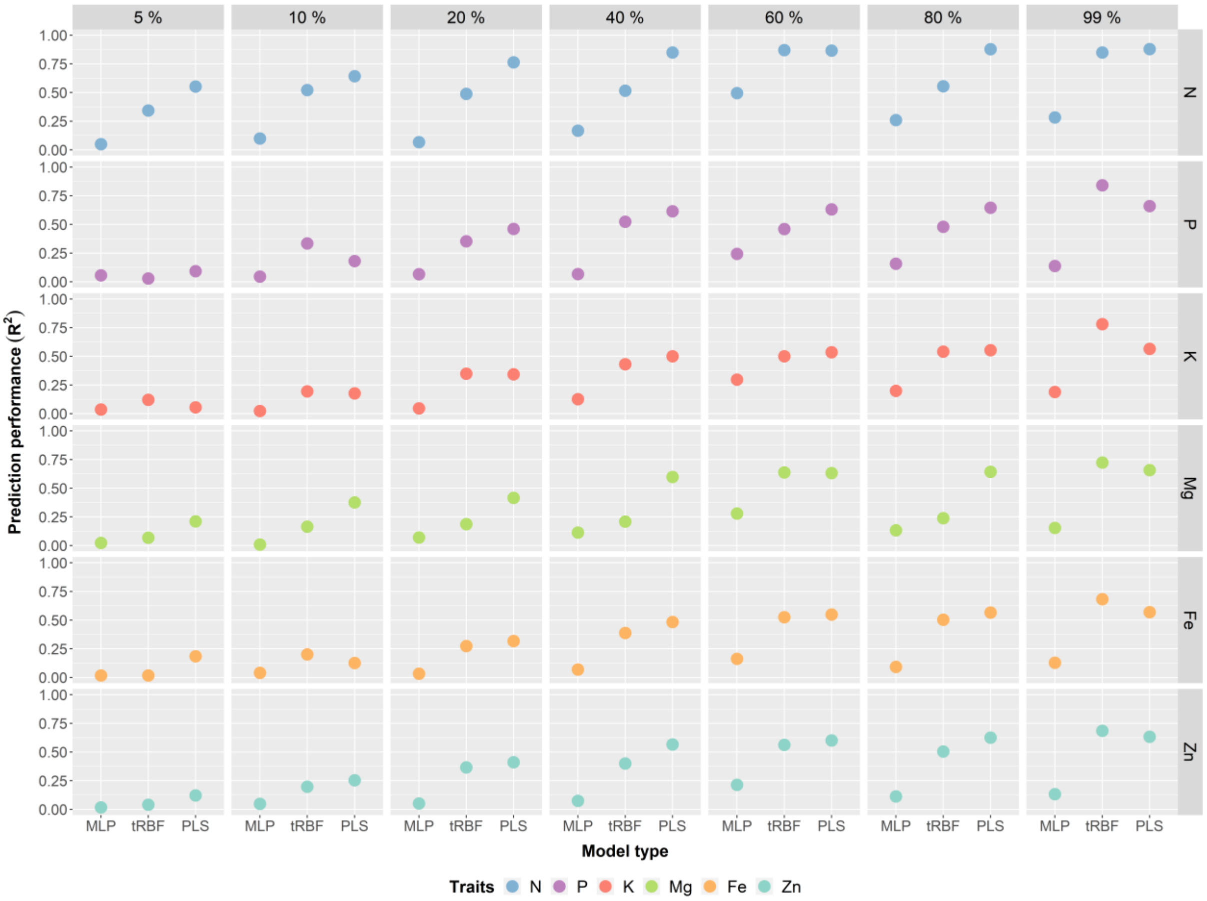

S6 Figure. Comparison of the investigated regression models in regard to prediction performance (R^2) across the four environments (DUN15, DUN16, HAL15 & HAL16) for each of the six nutrient traits (N, P, K, Mg, Fe & Zn) as rows and calibration set sizes (from 5% to 99%) as columns.
