# Supplementary material for: Optimizing the procedure of grain nutrient predictions in barley via hyperspectral imaging
Source: PLoS One. 2019 Nov 7;14(11):e0224491. doi: 10.1371/journal.pone.0224491 (PMC6837513; doi:10.1371/journal.pone.0224491)
Supplement: S9 Fig — (PDF) [file pone.0224491.s018.pdf]

S9 Figure.
Calibration set size comparison -Halle 2015 - Within traits

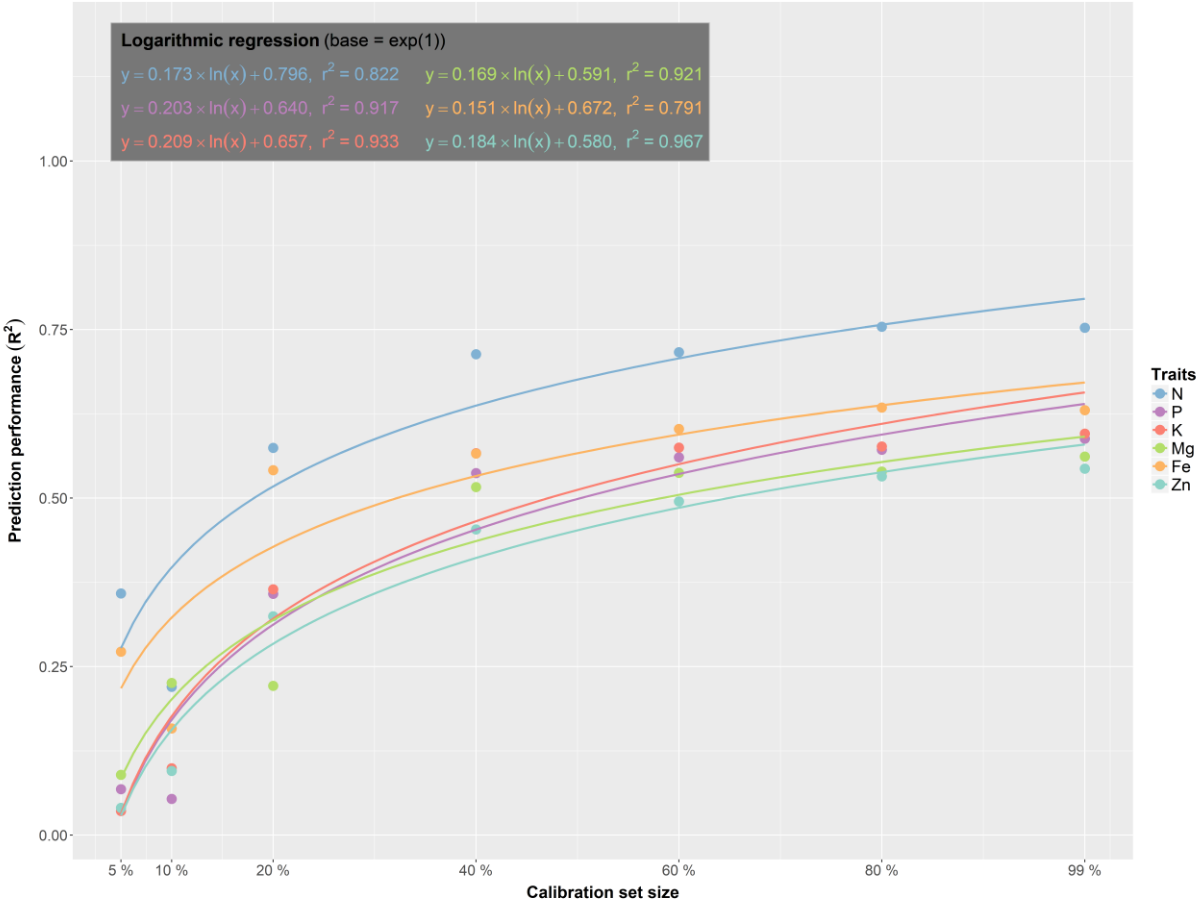

S9 Figure. Impact of calibration set size on prediction performance (R^2) in Halle 2015 for each of the six nutrient traits (N, P, K, Mg, Fe & Zn). A logarithmic function was fitted, which indicates the gain in prediction performance (R^2) with increasing calibration set sizes. The formulas of the six functions are shown in the upper left corner. The color of the dots and lines represents the traits.
