# Supplementary material for: Optimizing the procedure of grain nutrient predictions in barley via hyperspectral imaging
Source: PLoS One. 2019 Nov 7;14(11):e0224491. doi: 10.1371/journal.pone.0224491 (PMC6837513; doi:10.1371/journal.pone.0224491)
Supplement: S13 Fig — (PDF) [file pone.0224491.s022.pdf]

S13 Figure. Cost benefit analysis - With additional samples - Across environments - Within traits

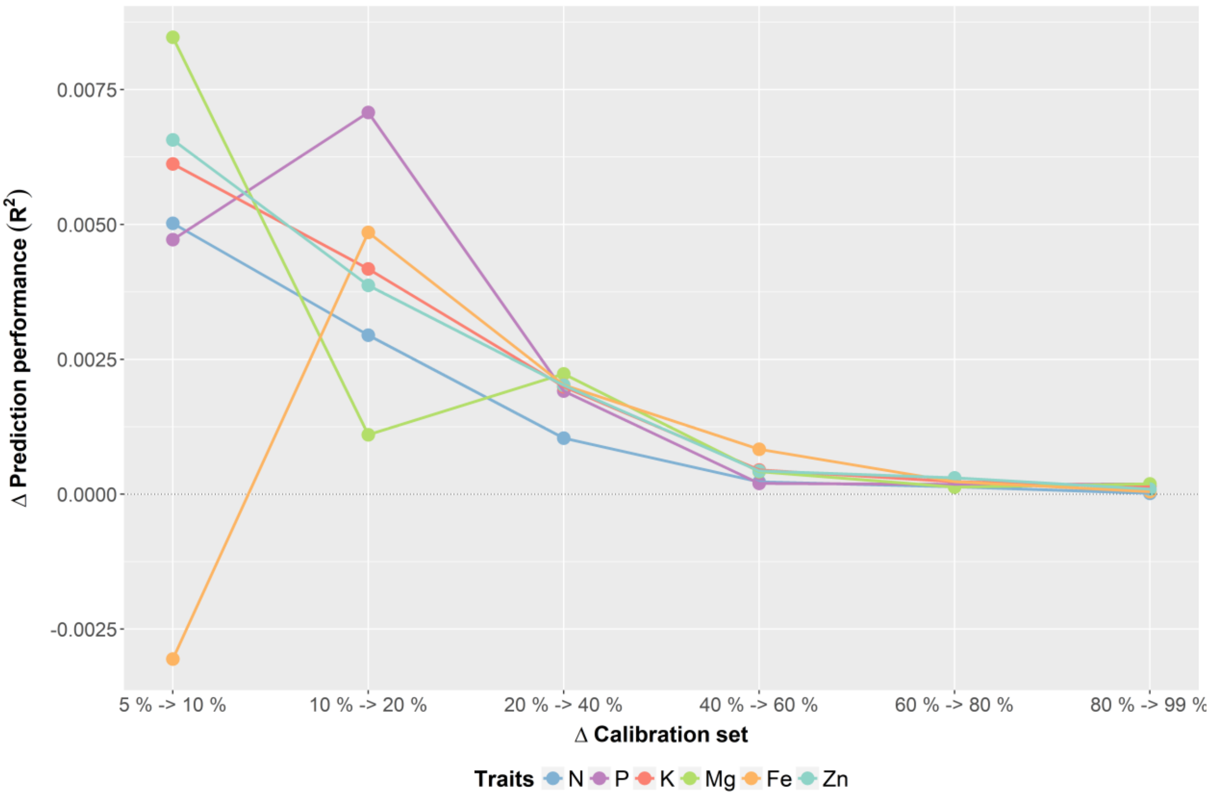

S13 Figure. Cost benefit analysis of prediction performance (R^2) gain by increasing calibration set size across the four environments (DUN15, DUN16, HAL15 & HAL16) and the six nutrient traits (N, P, K, Mg, Fe & Zn; indicated by different colors). The x-axis shows the six sample size increments of the calibration sets and the y-axis shows the respective gain or loss of prediction performance.
