# Supplementary material for: Optimizing the procedure of grain nutrient predictions in barley via hyperspectral imaging
Source: PLoS One. 2019 Nov 7;14(11):e0224491. doi: 10.1371/journal.pone.0224491 (PMC6837513; doi:10.1371/journal.pone.0224491)
Supplement: S14 Fig — (PDF) [file pone.0224491.s023.pdf]

S14 Figure. Relationship between trait value range covered by the calibration set and prediction performance (R^2) - Across environments - Within traits.

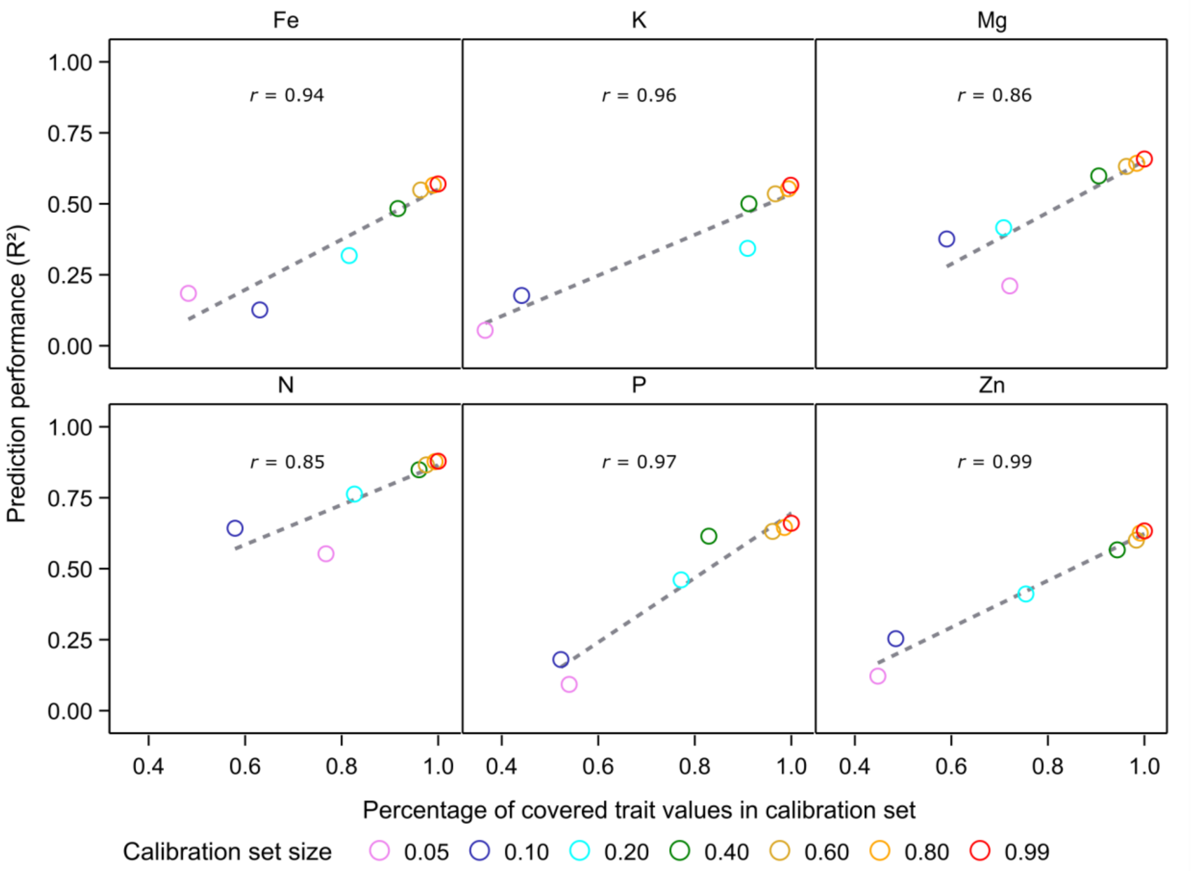

S14 Figure. Relationship between trait value range covered by the calibration set and prediction performance (R^2) across the four environments (DUN15, DUN16, HAL15 & HAL16) for each of the six nutrient traits (Fe, K, Mg, N, P & Zn). The x-axis shows the trait value range covered by the calibration set (calculated as $\frac{Max(CS)-Min(CS)}{Max(Total)-Min(Total)}$ and the y-axis shows the corresponding prediction performance (R^2). Pearson's correlation coefficients are given for each nutrient trait. Calibration set sizes are indicated by different colors.
