# Supplementary material for: Optimizing the procedure of grain nutrient predictions in barley via hyperspectral imaging
Source: PLoS One. 2019 Nov 7;14(11):e0224491. doi: 10.1371/journal.pone.0224491 (PMC6837513; doi:10.1371/journal.pone.0224491)
Supplement: S15 Fig — (PDF) [file pone.0224491.s024.pdf]

S15 Figure.
Model transferability - Within environments - Within traits

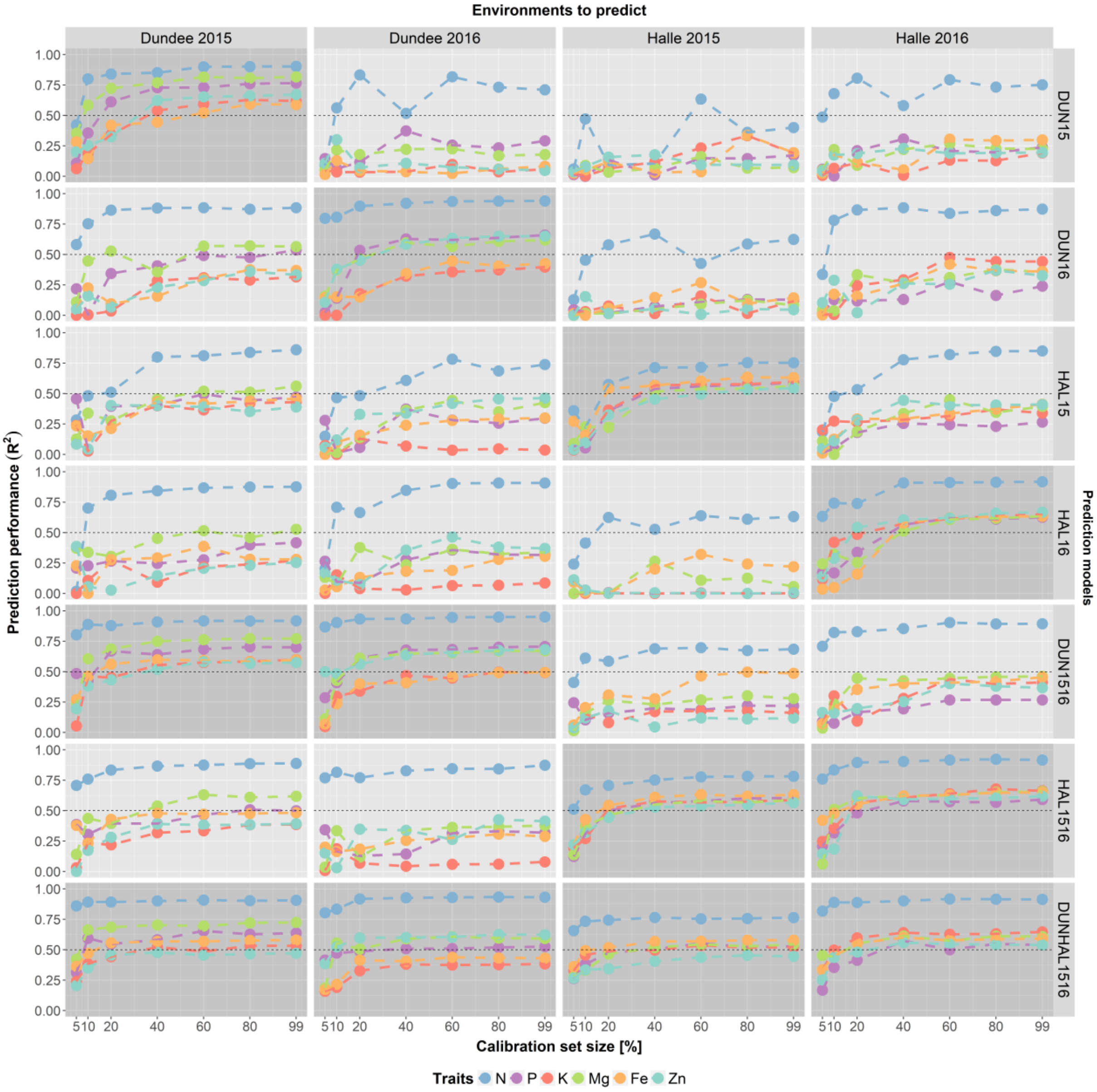

S15 Figure. Evaluation of model transferability to predict grain nutrients in each of the four environments (Dundee 2015, Dundee 2016, Halle 2015 & Halle 2016, shown as columns) for each of the six nutrient traits (N, P, K, Mg, Fe & Zn; indicated by different colors). Seven different prediction models (within each environment, across years, across environments; shown as rows) have been used to predict nutrient concentrations of the six traits in the four investigated environments The three types of prediction model compositions contain different numbers of samples: the four within environment models (DUN15, DUN16, HAL15 & HAL16) contain the simple number of samples of the respective environment, the two across years models (DUN1516 & HAL1516) the duplicated number of samples and the across environments model (DUNHAL1516) the quadruplicated number of samples.
